# Underserved groups could be better considered within population-based eye health surveys: a methodological study

**DOI:** 10.1016/j.jclinepi.2024.111444

**Published:** 2024-06-27

**Authors:** Lucy Goodman, Tulio Reis, Justine H. Zhang, Mayinuer Yusufu, Philip R. Turnbull, Pushkar Silwal, Mengtian Kang, Sare Safi, Hiromi Yee, Gatera Fiston Kitema, Anakin Chu Kwan Lai, Ian McCormick, João M. Furtado, Mostafa Bondok, Eric Lai, Sophie Woodburn, Matthew J. Burton, Jennifer R. Evans, Jacqueline Ramke

**Affiliations:** a School of Optometry & Vision Science, The University of Auckland, Auckland, New Zealand; b Division of Ophthalmology, Ribeir~ao Preto Medical School, University of S~ao Paulo, Ribeir~ao Preto, S~ao Paulo, Brazil; c International Centre for Eye Health, London School of Hygiene & Tropical Medicine, London, United Kingdom; d Centre for Eye Research Australia, Royal Victorian Eye and Ear Hospital, East Melbourne, Australia; e Department of Surgery (Ophthalmology), The University of Melbourne, Melbourne, Australia; f Beijing Tongren Eye Center, Beijing Tongren Hospital, Capital Medical University, Beijing Ophthalmology & Visual Science Key Lab, Beijing, China; g Ophthalmic Epidemiology Research Center, Research Institute for Ophthalmology and Vision Science, Shahid Beheshti University of Medical Sciences, Tehran, Iran; h Singapore Eye Research Institute, Singapore; i Department of Ophthalmology, University of Rwanda, Kigali, Rwanda; j Li Ka Shing Faculty of Medicine, The University of Hong Kong, Pok Fu Lam, Hong Kong; k Faculty of Medicine, The University of British Columbia, Vancouver, Canada; l National Institute for Health Research Biomedical Research Centre for Ophthalmology at Moorfields Eye Hospital NHS Foundation Trust and UCL Institute of Ophthalmology, London, United Kingdom

**Keywords:** Population based eye health surveys, Methodological review, Cross-sectional studies, Health equity analysis, Meta-epidemiology, Meta-research

## Abstract

**Objectives:**

In pursuit of health equity, the World Health Organization has recently called for more extensive monitoring of inequalities in eye health. Population-based eye health surveys can provide this information, but whether underserved groups are considered in the design, implementation, and reporting of surveys is unknown. We conducted a systematic methodological review of surveys published since 2000 to examine how many population-based eye health surveys have considered underserved groups in their design, implementation, or reporting.

**Study Design and Setting:**

We identified all population-based cross-sectional surveys reporting the prevalence of objectively measured vision impairment or blindness. Using the PROGRESS + framework to identify underserved groups, we assessed whether each study considered underserved groups within 15 items across the rationale, sampling or recruitment methods, or the reporting of participation and prevalence rates.

**Results:**

388 eye health surveys were included in this review. Few studies prospectively considered underserved groups during study planning or implementation, for example within their sample size calculations (*n* = ~ 5, ~1%) or recruitment strategies (*n* = 70, 18%). The most common way that studies considered underserved groups was in the reporting of prevalence estimates (*n* = 374, 96%). We observed a modest increase in the number of distinct PROGRESS + factors considered by a publication over the study period. Gender/sex was considered within at least one item by 95% (*n* = 367) of studies. Forty-three percent (*n* = 166) of included studies were conducted primarily on underserved population groups, particularly for subnational studies of people living in rural areas, and we identified examples of robust population-based studies in socially excluded groups.

**Conclusion:**

More effort is needed to improve the design, implementation, and reporting of surveys to monitor inequality and promote equity in eye health. Ideally, national-level monitoring of vision impairment and service coverage would be supplemented with smaller-scale studies to understand the disparities experienced by the most underserved groups.

## 1 Introduction

Equity in health has risen in prominence in recent decades and is now central to the aims of the United Nations’ Sustainable Development Goals (SDGs) [1]. Health equity is also central to efforts to realize Universal Health Coverage, which is a target within the SDGs (target 3.8) and a strategic priority for the World Health Organization [2]. In eye health, the World Health Organization’s World Report on Vision and the *Lancet Global Health* Commission on Global Eye Health both highlighted that in all parts of the world, there are population groups underserved by existing services, such as rural dwellers, women, Indigenous peoples, and nondominant ethnicity groups [3,4]. These reports also included calls for more evidence and action to address inequity, including better monitoring of health inequalities, which are measurable differences in health between population groups [5]. This monitoring can occur at eye health facilities, in terms of which population groups access services and the outcomes obtained, as well as through periodic population-based surveys.

Between 2000 and 2019, more than 250 population-based prevalence surveys of vision impairment were conducted globally [4]. These surveys have provided information that can be used by governments and their partners to strengthen eye health services to meet the needs of the population. The eye health need of underserved groups is sometimes assessed by conducting surveys exclusively within the underserved community (eg, within a refugee camp), but more often surveys have been conducted in a general population and the results disaggregated by population groups (eg, by sex/gender). The extent to which other methods have been used to include underserved population groups in eye health surveys is unknown.

In this study, we aimed to quantify how many population-based eye health surveys have considered underserved population groups in their design, implementation, and reporting. We have used the PROGRESS + framework [6] adopted by the Cochrane and Campbell Collaboration Equity Methods Group to systematically identify and classify underserved population groups into nine “factors” (Box 1).

## 2 Methods

We conducted and reported this study in accordance with the available guidance on the conduct and reporting of methodological studies [7,8]. We registered our protocol on Open Science Framework on 10 August 2022 (https://osf.io/cxdu4/) [9].

### 2.1 Eligibility criteria

We included all population-based cross-sectional surveys published since January 1, 2000, that estimate the prevalence of objectively measured distance or near vision impairment (and/or blindness) in a representative sample of people at the national, provincial/state, district, or subdistrict level in any country. To avoid excluding surveys that targeted minority or underserved groups, we included studies that estimated vision impairment within a reasonably small geographic sampling frame (eg, an entire population living in a village or a remote area) if they met other inclusion criteria. Studies exclusively involving children (<18 years) were excluded, as well as facility-based studies and studies that used self-reported vision loss. We also excluded studies reporting only prevalence estimates of eye conditions without reporting overall vision impairment (eg, prevalence of glaucoma). Studies reporting incidence were only considered if prevalence estimates from the baseline survey were available. We did not restrict our search based on language and included all studies identified by working with relevant translators. We excluded systematic reviews but reviewed the reference lists of relevant reviews to identify additional relevant studies.

### 2.2 Search strategy and study selection

The search strategy was developed by an experienced information specialist, drawing on the search used for a recent systematic review by the Vision Loss Expert Group [10]. Our search sought studies published on Medline, Embase, and SciELO databases between 1st January 2000 and the search date (initially July 11, 2022, and most recently November 15, 2023) (search strategy outlined in Supplementary Annex 1). We cross-checked our included studies against those identified in the previous Vision Loss Expert Group review [10]. As this is a methodological study of published literature, gray literature was not searched.

Screening was performed in Covidence (Veritas Health Innovation, Melbourne, Australia. Available at www. covidence.org). Two reviewers (from JR, JZ, JE, LG, PS, IM, MY, AL, and EL) independently screened i) title and abstracts, followed by ii) full text publications, against the inclusion criteria above, and conflicts were resolved through discussion. A third reviewer was involved when consensus could not be reached.

The results of some surveys were described in more than one publication and all eligible publications relating to the same survey (or group of closely related surveys, e.g., cohort studies) were grouped and considered together as a single “study”. We also included publications that described the survey protocol and retrieved additional publications (not identified from our search) that described detailed survey methods.

### 2.3 Study outcomes, data extraction, and analysis approach

Data extraction was performed in Covidence. The data extraction form was piloted by two investigators (LG, JR) on five studies and modified as required. Data extraction was then performed independently by two investigators (from LG, TR, JZ, MY, PT, PS, MK, HY, FK, AL, SS, IM, JF, MBo, EL, SW, JR), and discrepancies resolved by discussion, or with a third reviewer when necessary.

For each included study, we extracted the scope of the survey (multinational, national, or subnational), country/countries where the survey was conducted, year(s) of data collection, survey design (whether Rapid Assessment of Avoidable Blindness [RAAB] or its predecessor (Rapid Assessment of Cataract Surgical Services was used), sampling design (entire population/subsample), and the sample size. In reporting the characteristics of the included surveys, we summarized the survey locations within the Global Burden of Diseases (GBD) super regions (hereafter referred to as GBD regions) [11].

We assessed whether eye health surveys considered underserved groups according to 15 design or reporting items across five major study components—the study rationale, sampling methods, recruitment methods, reporting of participation and reporting of prevalence (Table 1). We made minor changes to the data extraction items from the protocol [9] to include any statistical comparisons of participation rates or prevalence estimates. For each item, we mapped any underserved groups identified to one or more of the PROGRESS + factors [6] outlined in Box 1. We calculated the number of studies that considered underserved groups: i) at least once in any of the 15 items, ii) at least once within each of the five components, and iii) within each of the individual 15 items. These data were reported as the number and percentage of all included studies. In the event of missing or unclear information, or an item not being applicable for a particular study, it was classified as not considering underserved groups. Statistical tests were performed in R (version 4.3.1, available from www.r-project.org/) using R Studio (2023.06.1, available from www.posit.co). The association between the median number of PROGRESS + factors and the publication year was calculated using linear regression weighted by the number of studies in each year. We compared the number of studies that considered underserved groups between national or multinational and subnational studies using chi-squared statistics, and these values were reported as the number and percentage of studies within each category.

## 3 Results

### 3.1 Characteristics of included studies

**Figure 1:**
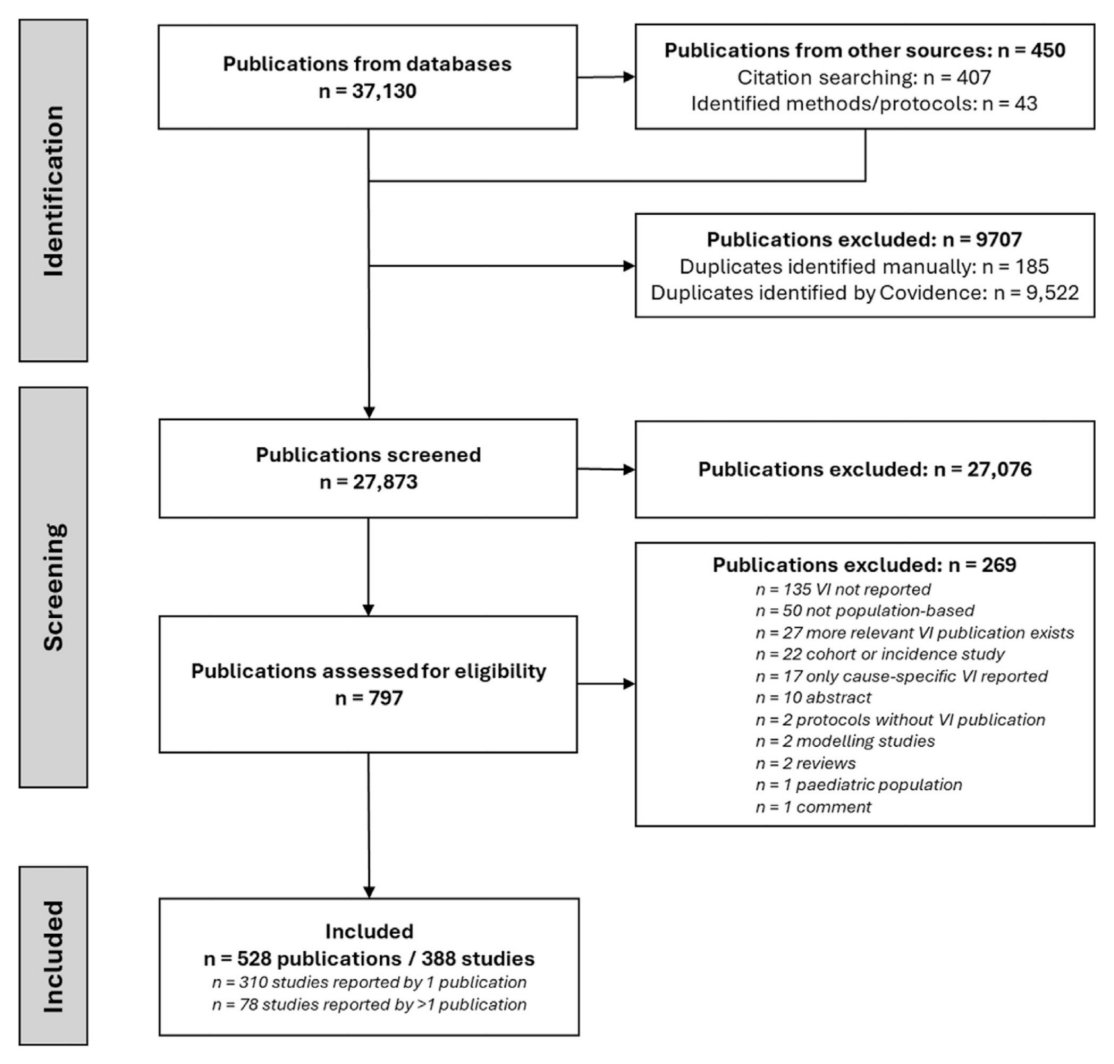
PRISMA flow diagram summarizing the screening and selection of studies included within this review. Note: individual “publications” reporting on the same (or closely related) population-based survey were grouped together into a single “study”.

Of the 37,580 publications retrieved from our search, we examined the full text of 797, and included 528 in the final analysis, reporting outcomes from 388 unique studies (Figure 1). Over a quarter of the 388 included studies (*n* = 106, 27%) were rapid assessments, most were conducted subnationally (*n* = 316, 81%), and were most often conducted in the GBD regions of South-East Asia, East Asia and Oceania (*n* = 107, 28%), sub-Saharan Africa (*n* = 87, 22%), or South Asia (*n* = 78, 20%) (Table 2). The most surveyed countries were China (*n* = 79, 19%), India (*n* = 53, 13%), and Nigeria (*n* = 31, 8%, Supplementary Figure 1). Forty-one percent of surveys (*n* = 159) were completed in 2010-2019, and most surveys recruited a sample between 1000 and 5000 participants (*n* = 228, 59%).

### 3.2 Do eye health surveys consider underserved groups?

Almost all studies (*n* = 387, > 99%) considered at least one PROGRESS + factor within any of the design or reporting items (median = 3, interquartile range 2-5 PROGRESS + factors considered per study), and this number modestly increased over the study period (+ 0.042 PROGRESS + factors per year, R^2^ = 0.19, *P* = .035, Supplementary Figure 2).

**Figure 2:**
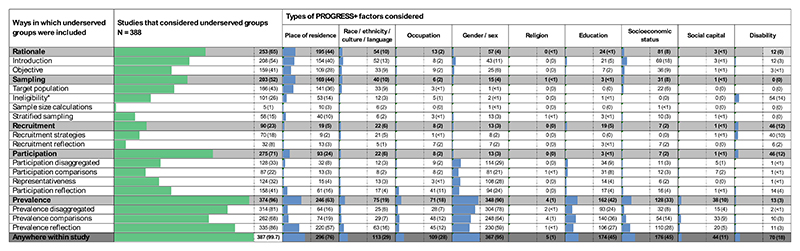
Proportion of eye health surveys that included underserved groups in their design or reporting of the study rationale, sampling, recruitment, participation, or prevalence estimates. Note: Gray rows indicate the number of studies that fulfill any of the criteria within that study component. The different types of underserved groups identified within each criterion were categorized within the PROGRESS + framework. The number of surveys is shown as a percentage of the 388 studies included in this review.

Overall, gender/sex was the PROGRESS + factor most frequently considered (*n* = 367, 95% of studies), followed by place of residence (*n* = 296, 76%); social capital (*n* = 44, 11%) and religion (*n* = 5, 1%) factors were considered infrequently (Figure 2). The “+ ” factor (hereafter referred to as “disability”) was considered in almost one in five studies (*n* = 70, 18%), although usually as an exclusion criterion (see ‘Sampling’ below).

### 3.3 How do eye health surveys consider underserved groups?

PROGRESS + factors were usually considered within items related to prevalence (*n* = 374, 96%) or participation (*n* = 275, 71%), followed by the survey rationale (*n* = 253, 65%) or sampling methods (*n* = 203, 52%), and least commonly within the recruitment methods (*n* = 90, 23%; gray rows Figure 2). The ways that studies considered underserved groups across these five components are described in more detail below, with examples of studies that prospectively considered underserved groups in study design outlined in Table 3.

#### 3.3.1 Study component 1: rationale

More than half (*n* = 208, 54%) of the included studies mentioned PROGRESS + factors in the introduction to the survey, including by describing disparities in eye health experienced by rural dwellers or people living in other underserved places of residence (*n* = 154, 40%), nondominant race, ethnicity, culture, or language (hereafter referred to as “race/ethnicity”) groups (*n* = 52, 13%), or women (gender/sex: *n* = 43, 11%), compared to more advantaged population groups (Figure 2).

#### 3.3.2 Study component 2: sampling

Forty-three percent of studies (*n* = 166) were conducted primarily on underserved population groups, and most frequently these were surveys of people living in underserved place of residence (Figure 2: *n* = 141, 36%). Examples of these included rural areas (*n* = 102, 26%), urban slums (*n* = 5, 1%), areas of conflict (*n* = 4, 1%), or people without stable housing (*n* = 3, <1%; data not shown) such as people visiting homeless shelters in Montreal (Table 3). Less often, the target population could be described by the race/ethnicity factor (*n* = 33, 9%; Table 2), and examples include people who identified with a nondominant ethnicity (*n* = 11, 3%), Indigenous people (*n* = 9, 2%), or tribal communities (*n* = 11, 3%; data not shown) such as in Andhra Pradesh in India (Table 3).

Fifteen percent of studies (*n* = 58) used stratified sampling to include specific population groups, often using the place of residence factor to ensure rural residents were sufficiently represented (*n* = 40, 10%; Figure 2). For example, the Melbourne Vision Impairment Project in Australia stratified the sample to represent urban Melbourne and rural Victoria (the state in which Melbourne is located; Table 3). Only five studies (1%) used more than one sample size calculation for different population groups within the same study (eg, the Australian National Eye Health Survey; Table 3). In contrast to these sampling methods that supported participation by underserved groups, more than one-quarter of included studies (*n* = 101, 26%) specifically excluded underserved population groups, usually by their place of residence (*n* = 53, 14%; e.g., institutionalized or people without housing) or by disability (*n* = 54, 14%; e.g., due to cognitive impairment; Figure 2).

#### 3.3.3 Study component 3: recruitment

Eighty percent of studies (*n* = 311) reported some details describing the recruitment strategies that were used (eg, to promote participation by raising awareness and building trust; data not shown). However, less than one-fifth (*n* = 70, 18%) described strategies that considered underserved groups (Figure 2). Studies most often considered underserved groups within the disability PROGRESS + factor (*n* = 40, 10%), usually by reporting the availability of home visits for people for whom the testing site was inaccessible. The race/ethnicity factor was also considered within some recruitment strategies (*n* = 21, 5%). For example, 17 studies (4%) provided a translator or emphasized the use of an appropriate language (data not shown). The Australian National Eye Health Survey and the Los Angeles Latino Eye Study are examples of surveys that considered the cultural appropriateness of their recruitment strategies (Table 3). Underserved groups within the gender/sex factor were occasionally considered within recruitment strategies (*n* = 8, 2%: Figure 2), with seven studies using female staff, and one providing childcare for participants (data not shown).

#### 3.3.4 Study component 4: participation

In the results section, about one-third of studies (*n* = 128, 33%) reported disaggregated participation rates (most frequently by gender/sex: *n* = 114, 29%; education: *n* = 34, 9%; or place of residence: *n* = 32, 8%), one-third described the representativeness of the study sample (*n* = 124, 32%), and one-fifth statistically compared participation rates (*n* = 87, 22%) within PROGRESS + factors (Figure 2). In the discussion, less than half of the studies discussed participation rates by underserved groups (*n* = 158, 41%), often by gender/sex (*n* = 94, 24%), place of residence (*n* = 61, 16%), or occupation (*n* = 41, 11%) factors.

#### 3.3.5 Study component 5: prevalence

PROGRESS + factors were frequently used in the reporting of vision impairment prevalence estimates (*n* = 314, 81% of studies; Figure 2). Prevalence estimates were most often disaggregated by gender/sex (*n* = 304, 78%), followed by level of education (*n* = 93, 24%) and place of residence (*n* = 64, 16%; e.g., rural vs urban locations). Two-thirds of the included studies (*n* = 262, 68%) used statistics to compare prevalence estimates, and this was again most common for gender/sex (*n* = 248, 64%), education (*n* = 140, 36%), and place of residence (*n* = 74, 19%). Almost all (*n* = 335, 86%) of the included studies mentioned PROGRESS + factors in the discussion section (eg, to explain their prevalence estimates), where the authors often referred to disparities in eye health by gender/sex (*n* = 230, 59%) or place of residence (*n* = 220, 57%), and less often by socioeconomic status (*n* = 110, 28%), education (*n* = 106, 27%), race/ethnicity (*n* = 63, 16%), or occupation (*n* = 45, 12%).

### 3.4 Does survey scope influence how underserved groups are considered?

The ways that studies considered underserved groups varied with the scale of the survey. Multinational or national surveys were more likely than subnational surveys to include underserved groups using stratified sampling (*n* = 18 of 72 multi/national studies, 25%, vs *n* = 40 of 316 subnational studies, 13%, χ^2^ = 7.03, *P* = .024, Supplementary Figure 3) or to disaggregate prevalence estimates in the reporting of the results (*n* = 68/72, 94% vs *n* = 246/316, 78%, χ^2^ = 10.46, *P* = .005). However, multinational or national surveys were less likely than subnational surveys to specifically target underserved population groups (*n* = 6/ 72, 8% vs *n* = 160/316, 51%, χ^2^ = 42.86, *P* < .001), to mention underserved groups within the introduction (*n* = 25/72, 35% vs *n* = 183/316, 58%, χ^2^ = 12.68, *P* = .002) or study objective (*n* = 20/72, 28% vs *n* = 139/ 316, 44%, χ^2^ = 6.37, *P* = .029), or to reflect on prevalence estimates across groups in the discussion (*n* = 52/72, 72% vs *n* = 283/316, 90%, χ^2^ = 14.94, *P* = .001).

## 4 Discussion

Our systematic methodological review has quantified how often researchers included underserved groups within the design, implementation and reporting of recent population-based eye health surveys. Our findings are reassuring in several ways given the need for reliable equity-relevant eye health data identified in the World Report on Vision [3] and the Lancet Commission on Global Eye Health [4]. First, almost half of the included studies were focused on an underserved population group as the target population, particularly people living in rural locations who tend to have worse access to health care compared to urban dwellers. Second, almost all studies considered PROGRESS + factors for at least one of the items we assessed. Third, we observed a small but significant increase in the median number of PROGRESS + factors considered in surveys since the year 2000.

Our findings also indicate that there is room for improvement if future population-based eye health surveys are to inform more equitable services. Aligned with the findings of a recent methodological review of general observational studies [12], we found that the included studies often considered underserved groups retrospectively. This was commonly achieved by disaggregating prevalence estimates, or by reflecting on increased prevalence of vision impairment or reduced access to eye health services among specific groups in the discussion. In contrast, very few studies prospectively calculated a separate sample size for an underserved group or described recruitment methods to promote participation by underserved groups. Concerningly, about a quarter of studies described actively excluding underserved groups (usually disabled people) from the survey. While we identified a handful of studies that included people without stable housing [27−29], the practice of enumerating households, or individuals resident in a household for at least 6 months, systematically excludes this population group.

The resolution passed by member states at the 74th World Health Assembly in 2021 [30] for integrated people-centered eye care has increased interest from many countries in methods to estimate service coverage at both the national level and across major population subgroups [31−33]. The national surveys included here almost all disaggregated prevalence estimates by at least one PROGRESS + factor to illustrate disparities between large population groups (eg, men and women, or geographic regions within a country). However, the national-scale means that smaller population groups, such as people without housing, incarcerated people, and refugees–which are arguably among the most underserved groups, are rendered invisible. Our results confirmed that subnational surveys often focus on generating prevalence estimates specifically within underserved (commonly rural) populations and can provide an important complement to national surveys. We also identified examples of surveys that used rigorous epidemiological methods to estimate prevalence exclusively among small, socially excluded groups, such as a Rohingya refugee camp in Bangladesh [24]. Generating evidence from these targeted surveys is the first step toward a socially inclusive health system [34]. Researchers wanting to better include underserved groups in large national or subnational level eye health surveys could increase the sample size (where budget allows) to ensure sufficient statistical power is achieved for meaningful comparisons between population subgroups. Other strategies include adapting recruitment methods to improve participation by underserved groups (eg, employing local health-care workers and/or female staff), and finding ways to include traditionally ineligible population groups such as people without housing [35]. Another approach is that taken in a RAAB survey in Papua New Guinea [23], where a sampling frame of four geographically distinct regions provided locally relevant information that was combined into national estimates of vision impairment and service coverage. Incorporating these methods will require commitment from researchers and funders, given the extended timeframes and higher resource costs required [35,36].

Our results must be interpreted in the context of several limitations. Incomplete reporting is a known challenge for authors of methodological reviews [37], and in eye health surveys [38], and while extensions to the STROBE reporting guidelines for eye health surveys [4] and equity [39] will assist future analyses, incomplete reporting will have impacted our results. To counter this, we erred toward including statements that acknowledged specific underserved groups regardless of the depth or direction of the discussion, which may have led to us overestimating the number of studies fulfilling some items (eg, the overrepresentation of women in some studies due to men being away from the home working when recruitment occurred). We also acknowledge that not all extraction items or PROGRESS + factors were equally relevant for each study. e.g., a study in a remote area is unlikely to stratify the sample or disaggregate results within the place of residence factor. We did not adjust our denominator to reflect this. We believe that by reporting absolute estimates for each item individually as well as grouping similar and mutually exclusive items together (eg, different sampling strategies), we have provided a robust estimate of how often studies are considering underserved groups. The data from this review is not yet available online, although our aim is to soon establish an ongoing, interactive database of these studies. Finally, as no official reporting guideline was available, we based our review design on the limited guidance available [7,8]. We believe that these limitations do not impact on the overall message of our results.

Our results highlight differences inherent in the objectives, design, and scale of the eye health surveys included that have implications for equity-focused planning. In the future, countries would ideally have access to national data describing vision impairment and service coverage among large population subgroups (eg, regions of the country, men and women, urban and rural dwellers) [3,4] while also having data to understand the extent of disparities experienced for the most marginalized and socially excluded groups, which would likely come from smaller-scale studies. Fortunately, regardless of the scale of eye health survey that researchers embark on, a range of strategies can be used to actively promote inclusion of historically underserved groups.

## Supplementary Material

**Supplementary data** Supplementary data to this article can be found online at https://doi.org/10.1016/j.jclinepi.2024.111444.

Supplementary Material

## Figures and Tables

**Table 1:** The criteria used to define whether a study considered underserved groups across the five study components and 15 items assessed

Design/reporting items	Yes – underserved groups were considered
Component 1: Rationale	
Introduction	• Described within-country population group/s who are underserved in relation to their eye health. This included statements of a specific population group that may experience:
	Increased prevalence or likelihood of experiencing vision impairment/blindness, cause-specific vision impairment/blindness. Reduced access to eye health services. Reduced participation in eye health surveys.
Objective	• Mentioned an underserved group within the aim or objective. Where the groups were underserved by their place of residence, the rationale had to explicitly describe the study location as underserved (e.g., describing the study location as ‘rural’ or a ‘disadvantaged area’).
Component 2: Sampling	
Target population	• The survey was conducted in a population group that was (predominantly) underserved or compared an underserved group with another population group (e.g., a survey of Indigenous vs non-Indigenous people).
Ineligibility^a^	• The survey explicitly excluded underserved group/s.
Sample size calculation	• The sample size calculation was performed separately for two or more population groups, at least one of which was underserved.
Stratified sampling	• Divided the population into strata for two or more population groups, at least one of which was underserved, before sampling from each.
Component 3: Recruitment	
Recruitment strategies	• Explicitly described recruitment methods that were different for underserved group/s compared to other populations; and/or • Applied recruitment methods that could reasonably be considered as targeting or enabling participation by underserved group/s, even if not explicitly stated (e.g., “We used female recruiters”).
Recruitment reflection	• Any self-reflection (positive or negative) in the discussion regarding the recruitment methods used and how these may have influenced participation by underserved groups.
Component 4: Participation	
Participation disaggregated	• Described the number of people who participated (as a proportion or percentage of the number invited) separately for two or more population groups, at least one of which was underserved.
Participation comparisons	• Compared whether underserved group/s were more or less likely to participate in the survey than other group/s using a statistical test (e.g., using logistic regression, odds ratios, including a *P* value etc.).
Representativeness	• Compared the number of participants in the sample to the number of people in the population, using data from a census (or similar) separately for two or more population groups, at least one of which was underserved.
Participation reflection	• Described how and/or why the participation rate did/did not differ in underserved group/s compared to other group/s; and/or • Described how the underserved group was overrepresented, or the advantaged group was underrepresented; and/or • Acknowledged that the study design influenced the participation rate for underserved group/s.
Component 5: Prevalence	
Prevalence disaggregated	• Reported the prevalence of vision impairment and/or blindness (as a number and/or percentage/proportion) separately for two or more population groups, at least one of which was underserved.
Prevalence comparisons	• Compared whether underserved group/s experience vision impairment and/or blindness at a different rate compared to other group/s using a statistical test (e.g., using logistic regression, odds ratios, including a P value etc.); and/or • Compared prevalence values between underserved group/s and other group/s by describing the prevalence with 95% confidence intervals (with or without P values).
Prevalence reflection	• Described how and/or why the prevalence of vision impairment did/did not differ in underserved population group/s compared to other group/s, with or without references to previous literature.


a The ineligibility item refers to studies that excluded PROGRESS + population groups, while all other items refer to methods that included PROGRESS + population groups.

**Table 2:** Characteristics of the included studies

Study characteristics	Total studies		Multinational		National		Subnational
	*n* (column %)		*n* (column %)		*n* (column %)		*n* (column %)
Number of studies	388		5		67		316
Survey design							
RAAB or RACSS	106 (27)		1 (20)		29 (43)		76 (24)
Other	282 (73)		4 (80)		38 (57)		240 (76)
Location (GBD Super-region)^a^							
South-East Asia, East Asia, & Oceania	107 (28)		1 (20)		12 (18)		94 (30)
Sub-Saharan Africa	87 (22)		3 (60)		10 (15)		74 (23)
South Asia	78 (20)		3 (60)		10 (15)		65 (21)
High Income	49 (13)		3 (60)		15 (22)		31 (10)
Latin America & Caribbean	32 (8)		1 (20)		12 (18)		19 (6)
North Africa & Middle East	31 (8)		0 (0)		4 (6)		27 (9)
Central Europe, Eastern Europe, & Central Asia	12 (3)		2 (40)		4 (6)		6 (2)
Sampling approach							
Sampled a subset of the population	344 (89)		5 (100)		67 (100)		272 (86)
Sampled entire population (e.g. complete population of one or more villages)	44 (11)		0 (0)		0 (0)		44 (14)
Sample size							
<1000	50 (13)		0 (0)		1 (1)		49 (16)
1000-5000	228 (59)		1 (20)		34 (51)		193 (61)
5000-10,000	52 (13)		1 (20)		9 (13)		42 (13)
>10,000	57 (15)		3 (60)		23 (34)		31 (10)
Not reported	1 (< 1)		0 (0)		0 (0)		1 (< 1)
Last year of data collection							
Before 2000	39 (10)		0 (0)		8 (12)		31 (10)
2000-2009	137 (35)		3 (60)		15 (22)		119 (38)
2010-2019	159 (41)		2 (40)		38 (57)		119 (38)
2020-2023	10 (3)		0 (0)		1 (1)		9 (3)
Not reported	43 (11)		0 (0)		5 (7)		38 (12)

RAAB, rapid assessment of avoidable blindness; RACSS, rapid assessment of cataract surgical services.

a The number of study locations is greater than the 388 included studies because five multinational studies were conducted in more than one Global Burden of Disease (GBD) super region.

**Table 3:** Examples of eye health surveys that prospectively considered underserved groups in their study design

Survey name/location	Features of the survey design that considered underserved groups	PROGRESS + factors
National Eye Health Survey, Australia [13–15]	Participants were sampled from specific areas where Indigenous people reside (Australian indigenous Geographic Classification Areas), and study sites were stratified by remoteness. Separate sample sizes were calculated for Indigenous and non-Indigenous Australians. A different age criterion was applied for Indigenous (40+ years) and non-Indigenous (50+ years) Australians to recognize the earlier onset and greater prevalence of eye disease, and lower life expectancy, of Indigenous Australians. The study was designed in collaboration with community elders and local health workers to ensure recruitment was culturally appropriate for Indigenous people. While the primary recruitment method was via door-knocking, Indigenous people were usually recruited via telephone from community lists, and via Aboriginal Health Clinics.	Place of residence; race/ethnicity/culture/language
Los Angeles Latino Eye Study, The United States of America [16–18]	The study area was selected due to a high proportion of the population being Latino, with a similar age distribution to that of Latinos across the United States. Reported findings from focus groups conducted prior to the survey. The focus groups addressed the cultural sensitivity of the questionnaires that would be administered during the survey and identified ways to improve the survey design to encourage participation. Recruitment strategies addressed the unique challenges faced by this population group. e.g., lack of trust was addressed by ensuring all staff were Latino and bilingual, and inconvenience of visiting the clinic away from the person’s home was addressed by providing free transport and childcare.	Race/ethnicity/culture/language
Melbourne Vision Impairment Project, Australia [19–22]	The sample was stratified by the participants’ place of residence: sampling areas were randomly selected from urban Melbourne and rural Victoria. In addition, nursing homes located near the urban clusters were randomly selected to ensure the institutionalized population was represented. Separate sample sizes were calculated for the noninstitutionalized (urban/rural) population and the institutionalized (nursing home) population.	Place of residence
Rapid Assessment of Avoidable Blindness, Papua New Guinea [23]	A nationally representative sample was achieved using RAAB methodology. To consider the geographic and environmental diversity across the country, PNG was divided into four regions (Highlands, Coastal, Islands, and National Capital District) and an equal number of participants sampled from each region.	Place of residence
Rapid Assessment of Avoidable Blindness Bangladesh [24]	The survey specifically targeted an underserved group. Eligible participants were forcibly displaced Myanmar Nationals (Rohingya refugees) residing in United Nations camps.	Place of residence
Rapid Assessment of Avoidable Blindness, Andhra Pradesh, India [25,26]	The survey specifically targeted an underserved group. Eligible participants resided in three selected tribal areas of Andhra Pradesh.	Place of residence; race/ethnicity/culture/language
Homeless shelters in Montreal, Canada [27,28]	These surveys specifically targeted an underserved group. Homeless shelters in the Montreal area were randomly selected, and a random sample of visitors were selected from each.	Place of residence


## Data Availability

Data will be made available on request.

## Box

**What is new?**

**Key findings**

- Studies often considered underserved groups in the reporting of prevalence estimates (eg, by disaggregating estimates between males and females). While many (often small-scale) studies specifically targeted underserved population groups, other prospective ways of including underserved groups—such as within their sampling or recruitment strategies—were less common.

**What this adds to what was known?**

- More effort is needed to include underserved groups within future eye health surveys.

**What is the implication and what should change now?**

- Where possible, future surveys could consider prospective methods of including underserved groups, such as increasing the sample size to allow for sufficiently powered comparisons between groups, or (where this is not feasible), considering recruitment strategies that enable participation by underserved groups.

## Box

**Box 1 Examples of underserved population groups within the PROGRESS D framework [6,12]**.

**Table:**

PROGRESS + factor	Examples of underserved groups
Place of residence^a^	Rural or remote areas Alternative, temporary, or noncommunity dwelling residences (e.g., asylum seekers, people without housing/rough sleepers, incarcerated, nursing homes, institutionalized) Disputed territories/areas of civil unrest
Race/ethnicity/culture/language	Nondominant ethnicity/language Indigenous
Occupation	Without full-time employment
Gender/sex	Women, transgender people
Religion	Religious minorities
Education	Nonliterate Lower education (e.g., years of schooling)
Socioeconomic status	Medium/low socioeconomic status
Social capital	Single/widowed/divorced (vs married) Marginalized/stigmatized Socially isolated
+^b^	Disabled

a The study location (eg, province, state, village etc) was not assigned to the ‘Place of residence’ factor unless it was explicitly described as underserved.

b The study location (eg, province, state, village etc) was not assigned to the ‘Place of residence’ factor unless it was explicitly described as underserved.

## Plain Language Summary

In all parts of the world, eye care services are less accessible to some groups of people (“underserved groups”). To address this inequity, the World Health Organization has suggested that countries regularly estimate the number of people who need eye care so that they can then design eye care services that better serve the needs of everyone in the population. The main way to get these estimates is by conducting a population-based survey. Whether previous surveys have included underserved groups is unknown, and exploring this was the aim of our study.

We identified all the eye health surveys published since the year 2000 and assessed how they included underserved groups–either in the way that they conducted the survey or the way that they reported the results. We observed that many small surveys specifically targeted underserved groups. We also found that surveys often considered underserved groups after the data was collected (eg, by comparing the number of people with vision loss in rural vs urban locations). However, most surveys were not designed to include underserved groups (eg, few surveys used specific methods to enable participation, such as survey staff speaking relevant languages or assistance with transport). Countries taking their first steps toward improved equity in eye health could focus on conducting surveys that consider underserved groups from the early survey planning stages.
