# Supplementary material for: Underserved groups could be better considered within population-based eye health surveys: a methodological study
Source: J Clin Epidemiol. Author manuscript; Available in PMC 2025 Oct 22. (PMC7618276; doi:10.1016/j.jclinepi.2024.111444)
Supplement: Supplementary Material [file EMS209634-supplement-Supplementary_Material.pdf]

Annex 1: Search strategy used to identify eye health surveys

MEDLINE

1. incidence/
2. prevalence/
3. epidemiology/
4. mortality/
5. morbidity/
6. age distribution/
7. sex distribution/
8. (epidemiology or incidence or prevalence or mortality).ti,ab.
9. or/1-8
10. exp blindness/
11. blindness.tw.
12. vision/
13. exp visual acuity/
14. (visual adj2 acuit$).tw.
15. vision, binocular/
16. (vision adj3 binocular).tw.
17. Vision, Low/
18. (low adj2 vision).tw.
19. night blindness/
20. presbyopia/
21. presbyopi$.tw.
22. Visually Impaired Persons/
23. ((vision or visual) adj3 impair$).tw.
24. (amaurosis or deaf-blind).tw.
25. or/10-24
26. 9 and 25
27. exp blindness/ep or exp blindness/mo
28. exp blindness/ and exp eye diseases/ep
29. vision, ocular/ep or vision, ocular/mo
30. Vision, Low/ep or vision, low/mo
31. Night Blindness/ep or night blindness/mo
32. Presbyopia/ep or presbyopia/mo
33. (population adj3 eye adj3 survey$).ti,ab.
34. (population adj3 vision adj3 survey$).ti,ab.
35. (population adj3 blindness adj3 survey$).ti,ab.
36. (population adj3 visual adj3 survey$).ti,ab.
37. or/27-36
38. 26 or 37
39. animals/ not humans/
40. (letter or comment or editorial).pt.
41. (clinical trial or controlled clinical trial).pt.
42. randomized controlled trial.pt.
43. (random$ adj3 trial$).ti.
44. review.pt.
45. case reports/
46. (case adj1 (report$ or series)).tw.
47. (mutation$ or genetic$ or genotyp$).ti.
48. or/39-47
49. 38 not 48
50. (Rapid adj2 Assessment adj2 Avoidable adj2 Blindness).tw.
51. 49 or 50
52. limit 51 to yr="2000 -Current"

Embase

1. incidence/
2. prevalence/
3. epidemiology/
4. mortality/
5. morbidity/
6. age distribution/
7. sex distribution/
8. (epidemiology or incidence or prevalence or mortality).ti,ab.
9. or/1-8
10. exp blindness/
11. blindness.tw.
12. vision/
13. exp visual acuity/
14. (visual adj2 acuit$).tw.
15. binocular vision/
16. (vision adj3 binocular).tw.
17. low vision/
18. (low adj2 vision).tw.
19. visual disorder/
20. night blindness/
21. refraction error/
22. myopia/
23. presbyopia/
24. presbyopi$.tw.
25. visual impairment/
26. ((vision or visual) adj3 impair$).tw.
27. (amaurosis or deaf-blind).tw.
28. or/10-27
29. 9 and 28
30. blindness/ep [Epidemiology]
31. exp blindness/ and exp eye diseases/ep
32. visual disorder/ep
33. low vision/ep
34. night blindness/ep
35. refraction error/ep
36. presbyopia/ep
37. myopia/ep
38. (population adj3 eye adj3 survey$).ti,ab.
39. (population adj3 vision adj3 survey$).ti,ab.
40. (population adj3 blindness adj3 survey$).ti,ab.
41. (population adj3 visual adj3 survey$).ti,ab.
42. or/30-41
43. 29 or 42

44. animals/
45. animal studies/
46. (amphibia or ape or bird or cat or cattle or chicken or dog or "ducks and geese" or fish or "frogs and toads" or goat or guinea pig or "hamsters and gerbils" or horse or monkey or mouse or "pigeons and doves" or "rabbits and hares" or rat or reptile or sheep or swine).tw.
47. or/44-46
48. human/
49. 47 not 48
50. 43 not 49
51. limit 50 to conference abstract status
52. 50 not 51
53. (letter or editorial or review).pt.
54. controlled clinical trial/
55. randomized controlled trial/
56. (random$ adj3 trial$).ti.
57. case report/
58. (case adj1 report$).tw.
59. (mutation$ or genetic$ or genotyp$).ti.
60. or/53-59
61. 52 not 60
62. (Rapid adj2 Assessment adj2 Avoidable adj2 Blindness).tw.
63. 61 or 62
64. limit 63 to yr="2000 -Current"

SCIELO

Search limited to topic – Ophthalmology
((ab:(incidence OR prevalence OR epidemiology))) AND (ab:(blindness OR vision OR "visual impairment" OR "refractive error"))

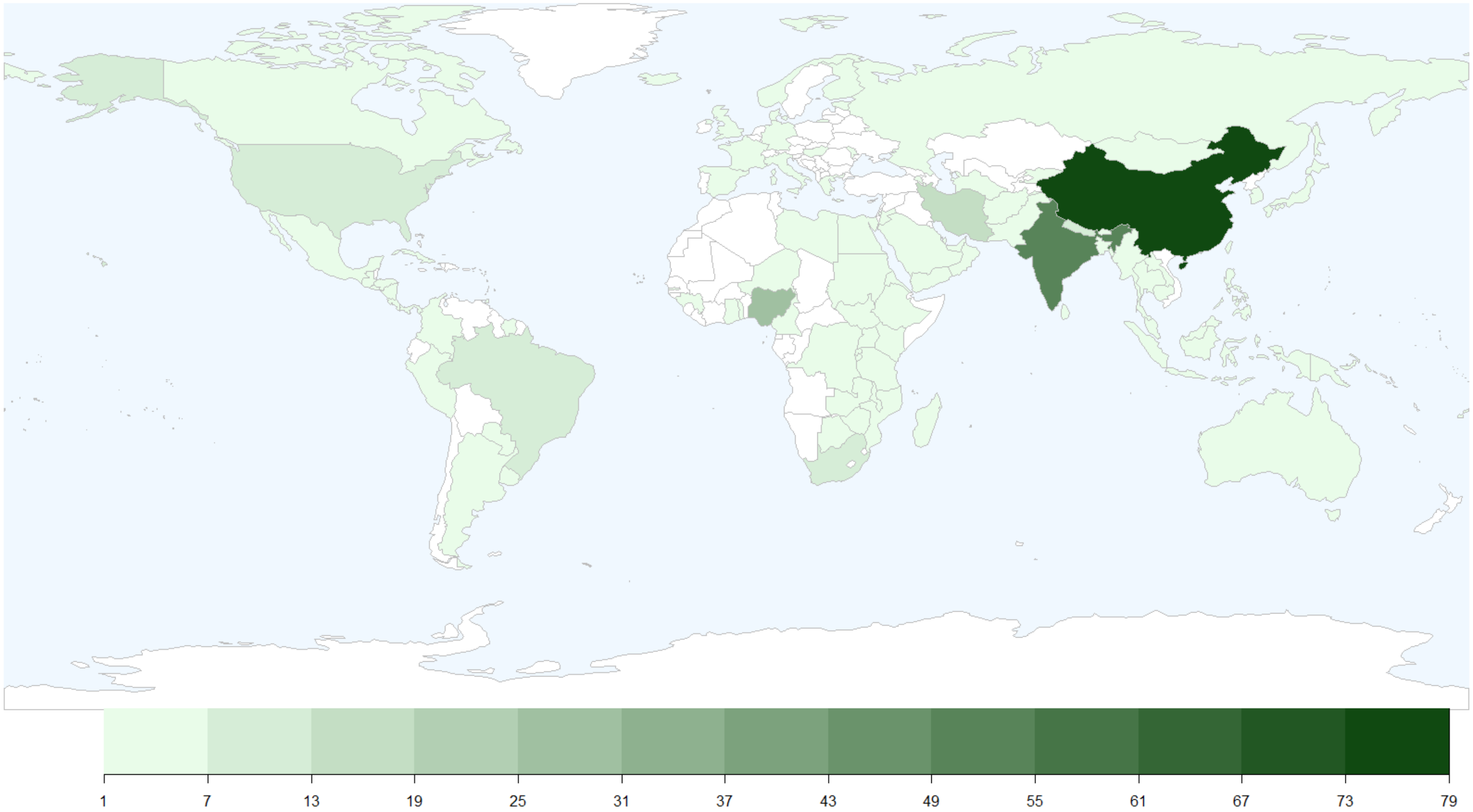

Supplementary Figure 1: World map illustrating the countries where the population-based eye health surveys included in this review were conducted.
Note: The number of countries shown is greater than the 388 included studies because multi-national surveys are shown across each included country.

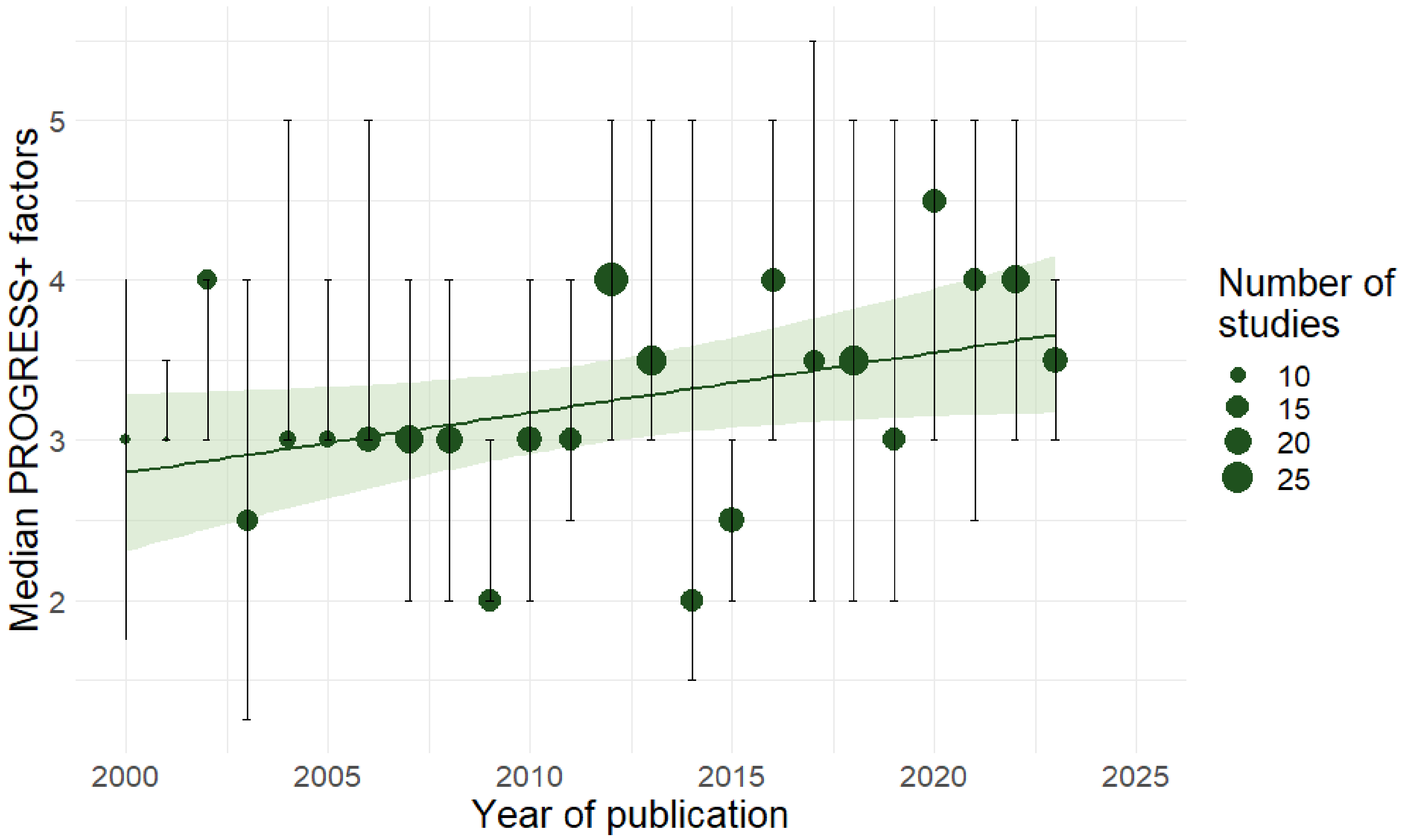

Supplementary Figure 2: Weighted linear regression of the number of PROGRESS+ factors considered within the included studies. Over the inclusion period, there was a modest increase in the median number of PROGRESS+ factors considered in each study (+0.042 per year).

Ways in which underserved groups were considered	National or Multi-national N = 72	Sub-national N = 316	p*
**Rationale**			-
Introduction	25 (35)	183 (58)	**0.002**
Objective	20 (28)	139 (44)	**0.029**
**Sampling**			-
Target population	6 (8)	160 (51)	**<0.001**
Ineligibility*	24 (33)	77 (24)	0.252
Sample size calculations	2 (3)	3 (<1)	0.322
Stratified sampling	18 (25)	40 (13)	**0.024**
**Recruitment**			-
Recruitment strategies	9 (13)	61 (19)	0.292
Recruitment reflection	6 (8)	26 (8)	0.977
**Participation**			-
Participation disaggregated	26 (36)	102 (32)	0.702
Participation comparisons	18 (25)	69 (22)	0.702
Representativeness	28 (39)	96 (30)	0.292
Participation reflection	28 (39)	130 (41)	0.778
**Prevalence**			-
Prevalence disaggregated	68 (94)	246 (78)	**0.005**
Prevalence comparisons	50 (69)	212 (67)	0.778
Prevalence reflection	52 (72)	283 (90)	**0.001**
**Anywhere within study**	**72 (100)**	**315 (99.7)**	-

Supplementary Figure 3: Comparison between the proportion of national and sub-national eye health surveys that include underserved groups within each of the 15 design or reporting items. Note: The 15 design or reporting items are listed within the five study components (grey rows). The number of surveys is shown as a percentage of the studies within the national (n= 72) or sub-national (n=316) categories.

**p-values are comparing the proportion of national and sub-national studies for each individual design or reporting item, where bold text indicates statistical significance at the 0.05 level.*

***The ineligibility item refers to studies that excluded PROGRESS+ population groups, while all other items refer to methods that included PROGRESS+ population groups.*
